# Size-controlled synthesis of cyclodextrin-capped gold nanoparticles for molecular recognition using surface-enhanced Raman scattering†

**DOI:** 10.1039/d1na00125f

**Published:** 2021-04-07

**Authors:** Koichiro Saito, Keegan McGehee, Yasuo Norikane

**Affiliations:** Research Institute for Advanced Electronics and Photonics, National Institute of Advanced Industrial Science and Technology (AIST) Higashi 1-1-1, Tsukuba Ibaraki 305-8565 Japan koichiro.saito@aist.go.jp; Department of Chemistry, Faculty of Pure and Applied Sciences, University of Tsukuba Ibaraki 305-8571 Japan

## Abstract

Cyclodextrin (CD)-capped gold nanoparticles (AuNPs) can be applied in sensing, catalysis, and self-assembly processes due to their molecular recognition ability. As the plasmon resonance of AuNPs depends on their size, the size-controlled synthesis of CD-capped AuNPs is essential for the development of these applications. Herein, we successfully synthesized β-CD-capped AuNPs with diameters of 24–85 nm using a seed-mediated growth method. The AuNPs were prepared using a β-CD as both the reducing agent and the capping agent. Harsh reagents such as NaBH_4_ and NaOH were not used. The size-controlled synthesis of β-CD-capped AuNPs was achieved by changing the amount of seed solution. We fabricated monolayers of β-CD-capped AuNPs by liquid–liquid interfacial self-assembly for application in surface-enhanced Raman scattering (SERS). The SERS intensity is significantly improved by using larger β-CD-capped AuNPs. In addition, we found that β-CDs can detect pyrene with higher sensitivity than α-CDs on the basis of the difference in molecular recognition ability between α-CDs and β-CDs.

## Abstract 2

Increased surface-enhanced Raman scattering (SERS) intensity and molecular-selective sensing were achieved by size-controlled synthesis of cyclodextrin-protected gold nanoparticles.

## Graphical abstract

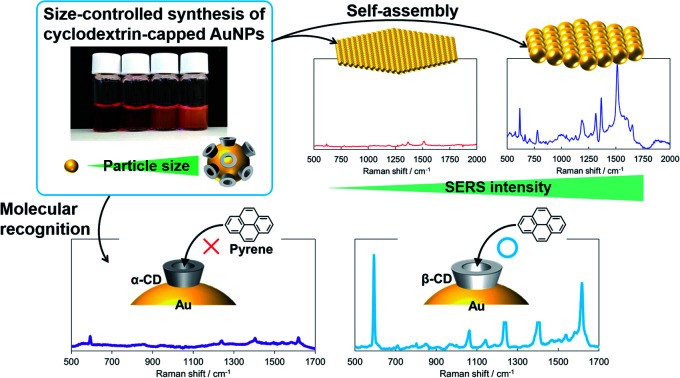

## Introduction

Gold nanoparticles (AuNPs) exhibit strong light absorption at specific wavelengths due to localized surface plasmon resonance. They have been applied in various research fields such as photonics,^1^ sensing,^2^ and energy conversion.^3,4^ In addition, the surface modification of plasmonic AuNPs can provide many attractive functions, such as biomedical performance,^5^ catalysis,^6^ and self-assembly.^7^ Macrocyclic molecules, such as cyclodextrins (CDs), pillararenes, and cucurbiturils, are remarkable candidates for the surface functionalization of AuNPs. For instance, CD-modified AuNPs have been employed in cascade catalysis,^8^ fluorescence sensing,^9^ surface-enhanced Raman scattering (SERS) sensing,^10^ and reversible self-assembly.^11^ CDs have a cavity with a hydrophilic exterior and a hydrophobic interior. Thus, various hydrophobic molecules can enter their cavity and be recognized by them. The size of the cavity, which determines the molecular recognition ability of CDs, becomes larger with an increase in the number of d-glucose units. CDs are named according to their sizes, where α-, β-, and γ-CDs are composed of six, seven, and eight d-glucose units, respectively. Each type of CD exhibits a different molecular recognition ability.^12^ β-CDs are often used for the surface functionalization of AuNPs because they can effectively recognize aromatic compounds. However, for such surface functionalization, ligand exchange using a thiolated β-CD is commonly performed,^10,11^ which is a time-consuming and expensive method.

Recently, it has been reported that β-CD-capped AuNPs can be easily synthesized using β-CD as a reducing agent. Oxidized β-CDs can then adsorb on the AuNP surface and act as a capping agent.^13^ This “green” synthesis method provides β-CD-capped AuNPs without using a strong base such as NaOH or NaBH_4_. β-CD-capped AuNPs, which are easily synthesized by such a green method, have a great advantage in practical use. However, the reported size of the resulting NPs is limited to approximately 20 nm. For applications such as fluorescence sensing^13^ and optical sensors,^14^ β-CD-capped AuNPs only need to exhibit plasmon resonance, which means that large-sized ones are not essential. Presumably this is one reason why the synthesis of large-sized β-CD-capped AuNPs has not been studied so far.

On the other hand, when applying β-CD-capped AuNPs to SERS, their size is an important factor, since the sensitivity of SERS increases with the size of the nanoparticles.^15^ Thus, it is required to develop a size-control method for β-CD-capped AuNPs. In addition, they have received a great deal of attention in SERS because selective molecular sensing due to the molecular recognition ability is enabled.^16–18^ Therefore, studying molecular-selective SERS sensing by using large-sized β-CD-capped AuNPs is also needed.

**Fig. 1:**
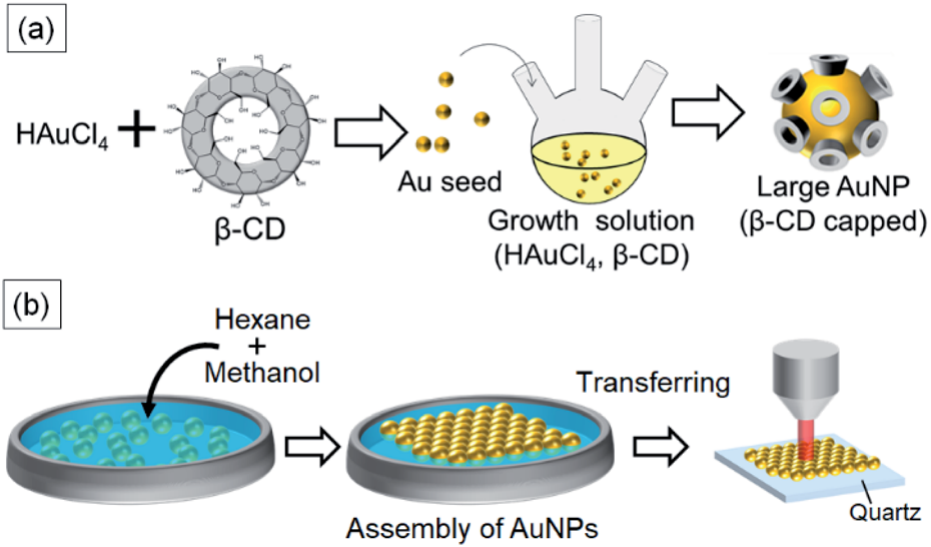
(a) Schematic illustration of seed-mediated growth of β-CD-capped Au nanoparticles. (b) Fabrication of a β-CD-capped AuNP monolayer using liquid–liquid interfacial self-assembly.

Herein, we report the size-controlled synthesis of α- and β-CD-capped AuNPs using CDs as the reducing agent and the capping agent (Fig. 1a). Using a seed-mediated growth method, the diameters of the β-CD-capped spherical AuNPs were controlled in the range of 24–85 nm. To the best of our knowledge, this is the first example of size-selective synthesis of β-CD-capped AuNPs with a diameter of about 80 nm, which are clearly larger than that of previously reported ones. Furthermore, we demonstrated that the β-CD-capped AuNPs assembled into a closely packed monolayer on a substrate *via* liquid–liquid interfacial assembly.^19^ We prepared monolayers of these β-CD-capped AuNPs with different sizes and applied them in SERS of rhodamine 6G (R6G) molecules. Basically, SERS intensity increases as the size of the NPs increases. As a result, a stronger SERS signal of R6G molecules was obtained from the monolayer consisting of larger β-CD-capped AuNPs (∼85 nm) than from that comprising smaller β-CD-capped AuNPs (∼24 nm). In addition, we detected pyrene molecules by use of the molecular recognition ability of β-CDs. We also fabricated α-CD-capped AuNPs of different sizes and assembled monolayers of those NPs. Comparing the SERS signals of pyrene shows the difference between the molecular recognition abilities of the α- and β-CD-capped AuNPs. The molecular recognition ability of the α-CD-capped AuNPs was clearly different from that of the β-CD-capped AuNPs. These size-controlled CD-capped AuNPs and self-assembled monolayers will contribute to the development of various applications such as SERS, biosensing, and catalysis.

## Experimental

### Materials

The α- and β-CDs, phosphate buffer solution (PBS, 0.1 M, pH = 7.0), pyrene and R6G were purchased from FUJIFILM Wako Pure Chemical Corporation. Chloroauric acid (HAuCl_4_·4H_2_O) and ethanol were obtained from Kishida Chemical Corporation. 4-Aminothiophenol (4-ATP) was procured from Tokyo Chemical Industries. All experiments were carried out using ultrapure water (>18 MΩ cm) prepared with a Direct-Q water purification system.

### Synthesis of CD-capped seed AuNPs

The β-CD-capped seed AuNPs were synthesized according to a previously reported method^13^ with modifications. Pure water (35 mL), 0.1 M PBS (5 mL), 0.01 M β-CD aqueous solution (10 mL), and 0.01 M HAuCl_4_·4H_2_O aq. (1 mL) were mixed in a flask. The reaction mixture was stirred and refluxed for 30 min. For the synthesis of the α-CD-capped seed AuNPs, 37.5 mL of pure water, 2.5 mL of 0.1 M PBS, 10 mL of 0.01 M α-CD aq., and 1 mL of 0.01 M HAuCl_4_ aq. were mixed in a flask and refluxed for 30 min.

### Seed-mediated synthesis of large CD-capped AuNPs

The large β-CD-capped AuNPs were fabricated *via* seed-mediated synthesis. Pure water (47.5 mL), 0.1 M PBS (2.5 mL), 0.01 M β-CD aq. (100 μL), and 0.01 M HAuCl_4_ aq. (100 μL) were mixed in a flask as the growth solution. A certain amount of seed solution (2 mL, 0.5 mL, and 0.2 mL) was added to the reaction mixture followed by refluxing for 1–2 h. For the synthesis of the α-CD-capped AuNPs, 48.75 mL of pure water, 1.25 mL of 0.1 M PBS, 100 μL of 0.01 M α-CD aq., and 100 μL of 0.01 M HAuCl_4_ aq. were mixed in a flask as the growth solution. In the same manner, 2 mL, 0.5 mL, and 0.2 mL of seed solution were added to the reaction mixture followed by refluxing for 1–2 h. To purify the CD-capped AuNPs, the solution was centrifuged, and the precipitate was redispersed in pure water.

### Fabrication of CD-capped AuNP monolayers

A colloidal solution of CD-capped AuNPs (500 μL) was added onto a polytetrafluoroethylene dish dropwise. Hexane (800 μL) was added to the dish to form an oil–water interface. Subsequently, 800 μL of methanol was rapidly added to the resulting solution, leading to the formation of a CD-capped AuNP monolayer at the interface. A quartz substrate was cleaned with UV ozone for 30 min. The CD-capped AuNP monolayers were deposited on these substrates by placing the substrates just below the interface.

### Molecular adsorption on CD-capped AuNP monolayers

To investigate the effect of particle size on the strength of the SERS signal, R6G was used as a model analyte. 10 μL of 10^−5^ M R6G ethanol solution was added dropwise onto the monolayer-deposited quartz substrate and dried under ambient conditions. The area of the substrates was about 1 cm^2^. The normal Raman measurement for R6G was carried out by drop casting 10 μL of 10^−2^ M R6G ethanol solution on a bare quartz substrate. The area of the substrate was also 1 cm^2^.

For studying host–guest interactions between the CD and a target molecule, pyrene and 4-aminothiophenol were used. Each target molecule was adsorbed on the CD-capped AuNP monolayer by immersing the monolayer-deposited quartz substrate in a solution containing the target molecule. 2 × 10^−7^ M pyrene aq. solution was prepared by adding 10 μL of a 2 × 10^−4^ M pyrene ethanol solution to 9.99 mL of pure water. After immersing the monolayer-deposited quartz substrate in the as-prepared solution for 2 h, the resulting substrate was rinsed with pure water and dried under a nitrogen stream. In the case of 4-ATP, the monolayer-deposited substrate was immersed in a 1 μM 4-ATP ethanol solution for 2 h. After immersion, the resulting substrate was rinsed with ethanol and pure water and then dried under a nitrogen stream.

### Characterization

Transmission Electron Microscopy (TEM) images were taken using a Philips CM200UT. The CD-capped AuNP monolayers deposited on quartz were investigated by scanning electron microscopy (SEM, JEOL JSM6700F). The average size of the CD-capped AuNPs was calculated using Fiji image processing software (ImageJ). The hydrodynamic diameters and the zeta-potentials of the AuNPs were measured by using a dynamic light scattering (DLS) and zeta-potential measurement system (Zetasizer NanoZS, Malvern Instruments). Optical extinction spectra of the CD-capped AuNP dispersion and CD-capped AuNP monolayer were obtained using a UV-vis spectrometer (JASCO V670). SERS was carried out using a Raman spectrometer (PerkinElmer Raman station 400F). The excitation wavelength was 785 nm, and the integration time was 300 s.

## Results

### Size-controlled synthesis of CD-capped AuNPs

**Fig. 2:**
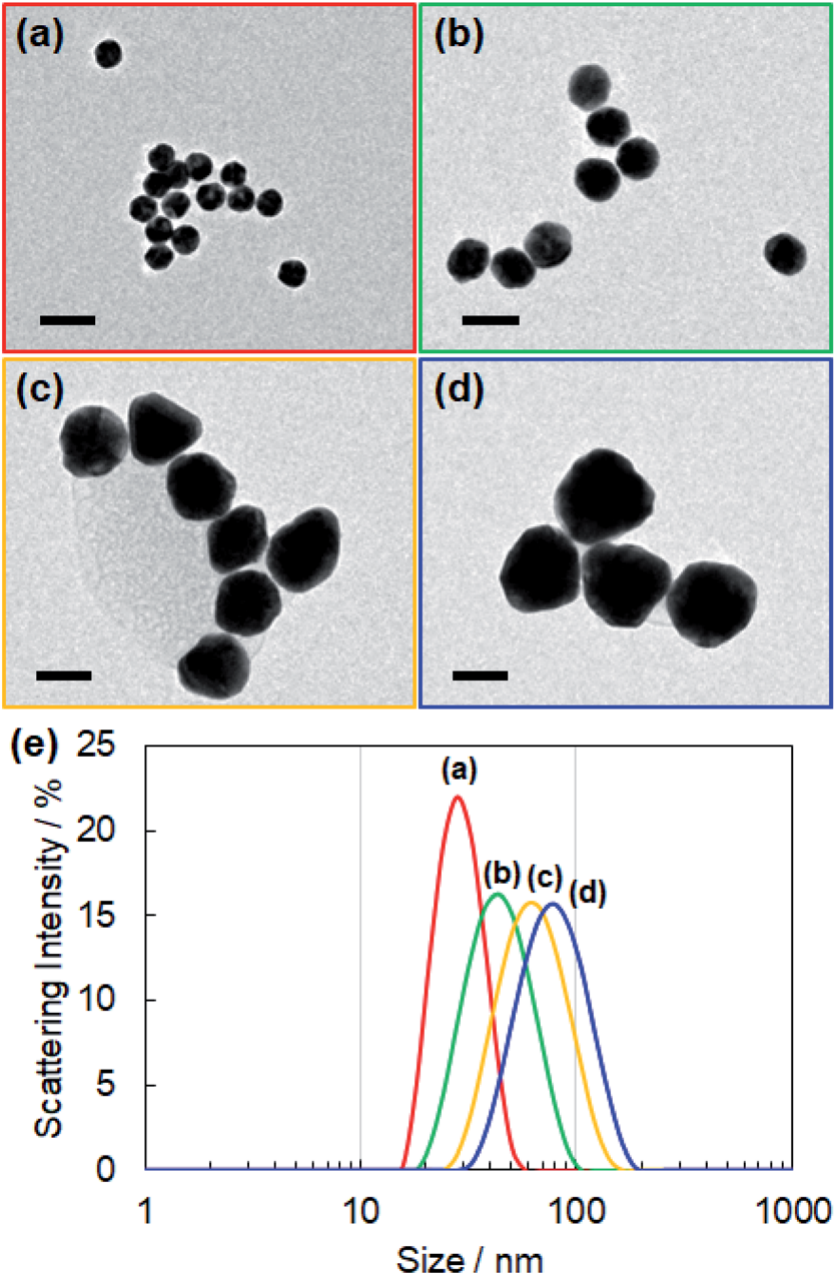
TEM images of the β-CD-capped AuNPs with average diameters of (a) 24, (b) 41, (c) 63, and (d) 85 nm, respectively. The scale bar is 50 nm for all the TEM images. (e) Size distribution of β-CD-capped AuNPs for each size measured by DLS.

**Fig. 3:**
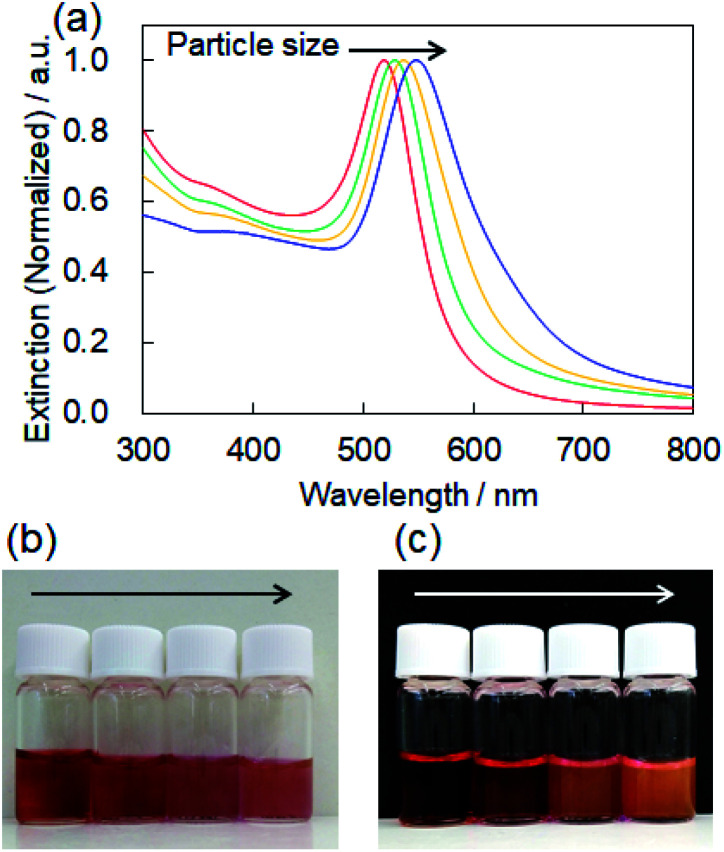
(a) Extinction spectra of the β-CD-capped AuNPs dispersed in an aqueous solution corresponding to Fig. 2a–d. Photographs of the (b) transmitted and (c) scattering color of the β-CD-capped AuNPs dispersed in the aqueous solution.

For controlling the size of the CD-capped AuNPs, we modified the seed-mediated growth method (Fig. 1a), which has been employed for the synthesis of citrate-capped AuNPs.^20–23^ The seed AuNPs were synthesized according to a previously reported method.^13^ The average diameter of the AuNPs was determined to be 24 ± 1.5 nm by TEM (Fig. 2a). The hydrodynamic diameter measured by DLS was 28 nm. The zeta-potential value of the AuNPs was −44 mV. The as-synthesized seed AuNPs were grown by refluxing them in a growth solution containing a β-CD, HAuCl_4_, and PBS. The size control of the β-CD-capped AuNPs was achieved by varying the amount of seed solution. As shown in the TEM images (Fig. 2b–d), the particle size increased with a decrease in the amount of seed solution. The diameters of the three corresponding AuNPs were determined to be 41 ± 3.5, 63 ± 7.9, and 85 ± 12.2 nm by TEM. The dispersions of β-CD-capped AuNPs with different particle size distributions were successfully obtained. The particle size distributions measured from the TEM images are shown in Fig. S1a–d (ESI).† The hydrodynamic diameters of these AuNPs calculated by DLS measurements were 37, 55, and 74 nm, which were in agreement with the TEM results. The size distribution obtained from DLS (Fig. 2e) also supported the achievement of size-controlled synthesis. The zeta-potential values of the AuNPs ranged from −44 to −48 mV, similar to the zeta-potential of the seed AuNPs. In this method, the amounts of gold precursor and β-CD were fixed throughout, whereas the amount of seed solution was decreased, leading to larger β-CD-capped AuNPs. The size and zeta-potential of AuNPs corresponding to each amount of seed solution are shown in Table S1 (ESI).† The UV-vis spectra of the β-CD-capped AuNPs were recorded in an aqueous solution. The peak red-shifted from 519 to 548 nm with an increase in the size of the β-CD-capped AuNPs (Fig. 3a). It is known that with an increase in the diameters of the NPs, the resonance wavelength of spherical plasmonic NPs red-shifts and the scattering intensity increases.^24,25^ As shown in Fig. 3b and c, although the transmitted colour of the β-CD-capped AuNPs changed from ruby red to purple with an increase in the particle size, orange scattering became stronger.

The size control of the α-CD-capped AuNPs was also attempted. However, the seed particles did not grow under the same conditions as those used for the β-CD-capped AuNPs. By decreasing the concentration of PBS, size-controlled α-CD-capped AuNPs were successfully obtained. The size distributions measured by DLS and their extinction spectra are shown in Fig. S2 and S3,† respectively. The size and zeta-potential of AuNPs corresponding to each amount of seed solution are also shown in Table S1.† The growth solutions of both α- and β-CD-capped AuNPs had the same pH (∼7), which was maintained by using PBS. Therefore, pH was not the cause of the failed growth of α-CD-capped AuNPs. It is possible that the relatively higher concentration of PBS induced the aggregation of the α-CD-capped seed AuNPs during the reaction.

### Fabrication of β-CD-capped AuNP monolayers

For SERS sensing, we prepared two-dimensional monolayers of β-CD-capped AuNPs (Fig. 1b). An organic solvent-mediated liquid–liquid interfacial assembly method was employed for the preparation of these monolayers.^19^ When water is mixed with an organic solvent such as hexane, an organic–aqueous interface is formed between the two liquid phases. If the water layer contains dispersed NPs, the addition of a polar solvent, such as methanol, decreases their surface charge. It has been reported that the adsorption of the polar solvent molecules on the surface of NPs causes a slight decrease in the surface charge density.^19,26^ In addition to this effect, the NPs are adsorbed at the organic–aqueous interface to minimize the interfacial energy at the interface. As a result, a self-assembled monolayer of NPs is formed at the interface.^19,27^ Citrate-capped AuNPs are often used in this method. As the surface of the β-CD-capped AuNPs is negatively charged, similar to that of citrate-capped AuNPs,^20^ the liquid–liquid interfacial assembly technique was applicable. Thus, using the above method, we successfully fabricated β-CD-capped AuNP monolayers on a quartz substrate using β-CD-capped AuNPs of various sizes.

**Fig. 4:**
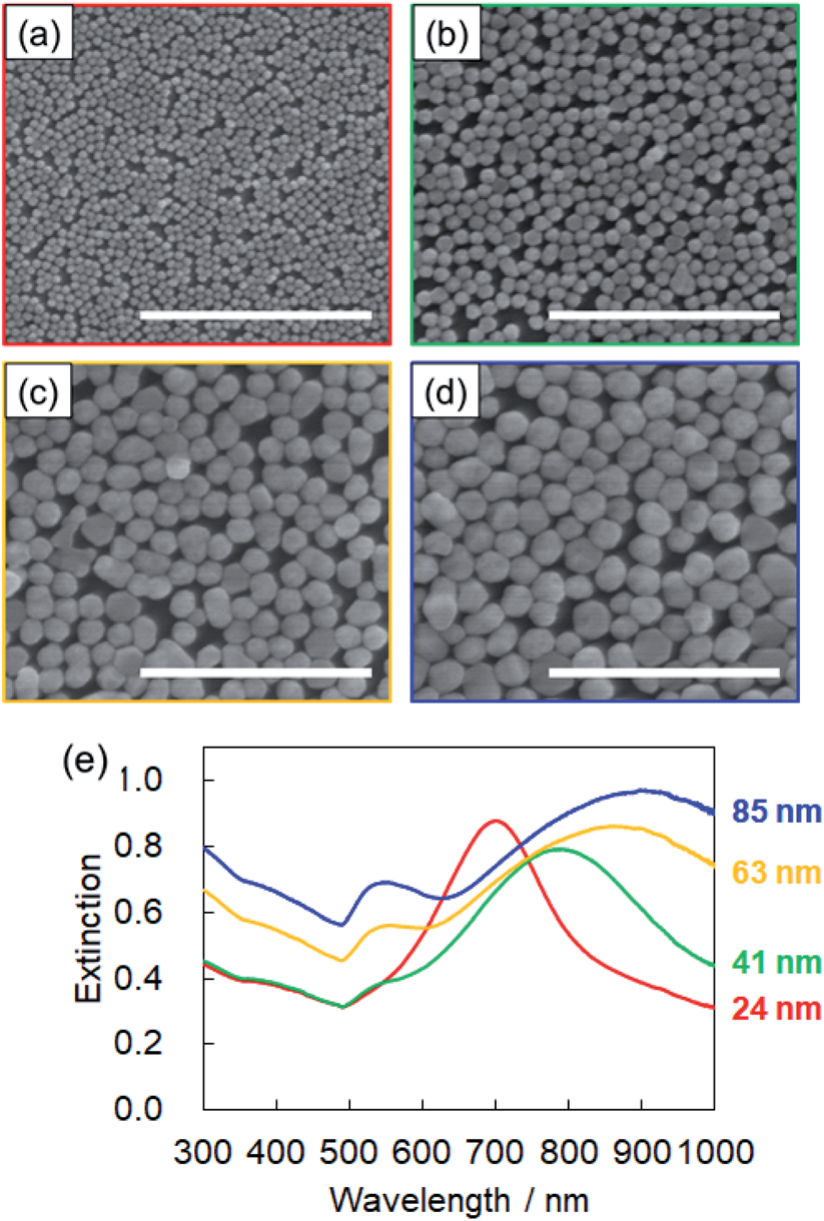
(a) SEM images of β-CD-capped AuNP monolayers with diameters of (a) 24, (b) 41, (c) 63, and (d) 85 nm, respectively. The scale bar is 500 nm for all the SEM images. (e) Extinction spectra of the β-CD-capped AuNP monolayers of each size.

The SEM images of the monolayers deposited on quartz substrates are shown in Fig. 4a–d. The β-CD-capped AuNPs of all sizes are closely packed on the quartz. To the best of our knowledge, this is the first time that the fabrication of two-dimensional monolayers of large (∼80 nm in diameter) CD-capped AuNPs has been achieved. Fig. 4e shows the extinction spectra of the monolayers. The peak of the CD-capped AuNP monolayer with a 24 nm diameter was located at 700 nm, which was significantly red-shifted relative to the peak at 519 nm for the original β-CD-capped AuNPs dispersed in water. When two or more NPs are close to each other, their plasmon resonances couple.^28^ This plasmon coupling effect causes the redshift of the peak of the monolayer, which is beneficial for SERS sensing using a near-infrared laser. In the case of the β-CD-capped AuNP monolayer with an 85 nm diameter, a broad peak appeared at an even longer wavelength (900 nm). The successful fabrication of two-dimensional monolayers of β-CD-capped AuNPs *via* the simple preparation process employed herein proved that a functional plasmonic monolayer can be obtained without ligand exchange.

### Increased SERS signal due to larger AuNPs

We utilized the fabricated monolayers for SERS sensing. In SERS, the intensity of the Raman signals from the molecules near the surfaces of metal nanostructures is significantly enhanced due to the generation of electromagnetic fields by plasmon resonance near the metal surface. The intensity of electromagnetic fields becomes even stronger in the assembled layers due to plasmon coupling, and the gaps between NPs are called “hot spots.” As two-dimensional assemblies of plasmonic NPs have a large number of hot spots,^29^ they can realize high Raman sensitivity. Therefore, they have attracted considerable scientific interest in recent years.^30–32^ Herein, two-dimensional monolayers consisting of β-CD-capped AuNPs of different sizes were applied in SERS of R6G, which is commonly used as a model analyte to evaluate the SERS sensitivity.

**Fig. 5:**
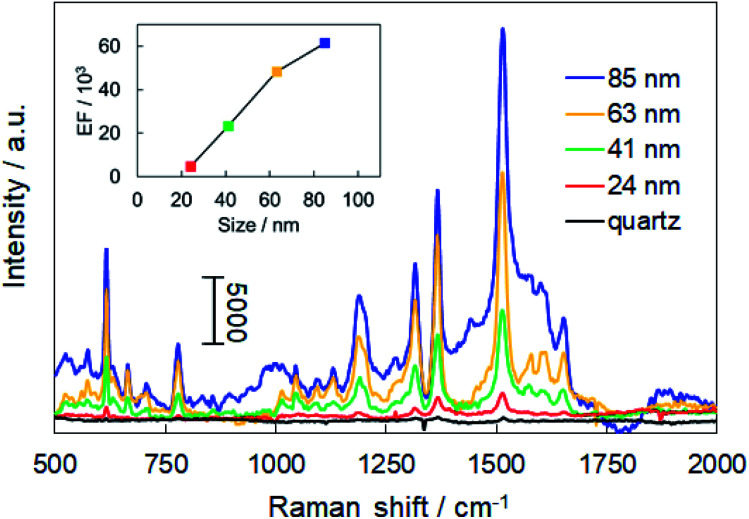
SERS spectra of R6G deposited on AuNP monolayers of each size. For comparison, the normal Raman spectrum of R6G deposited on a quartz substrate is also shown. The inset shows the EFs for AuNP monolayers of each size.

The Raman spectra of R6G adsorbed on each of the AuNP monolayers and a bare quartz substrate are shown in Fig. 5. The Raman signal from the AuNP monolayers was so strong that multiple clear peaks were observed. The peaks at 616, 778, and 1188 cm^−1^ are assigned to the in-plane xanthene ring deformation of R6G and those at 1315, 1366, 1513, and 1651 cm^−1^ are assigned to xanthene ring stretching modes.^33,34^ On the other hand, the Raman signal of R6G deposited on the quartz substrate was very weak despite the use of a 1000 times higher concentration of R6G solution. This result demonstrates that the monolayer of self-assembled β-CD-capped AuNPs has SERS activity. The enhancement factor (EF) for each AuNP monolayer was estimated by using the following eqn (1):^35,36^1
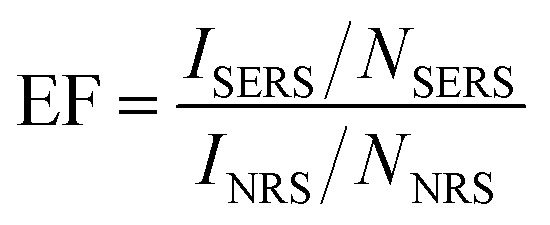
where *I*_SERS_ is the intensity of a Raman signal obtained from each AuNP monolayer and *I*_NRS_ is the intensity of a normal Raman signal obtained from a quartz substrate. The intensity of the peak at 616 cm^−1^ was used for the estimation of the EF values. *N*_SERS_ and *N*_NRS_ are the number of R6G molecules excited by laser irradiation in the area of the laser focus spot on each AuNP monolayer and on the quartz, respectively. In this experiment, the area of the substrate and the amount of dropped R6G solution were fixed for all the substrates. On the other hand, the concentration of R6G solution used for the quartz substrate was 1000 times higher than that used for each AuNP monolayer. The calculated EFs for AuNP monolayers with diameters of 24, 41, 63, and 85 nm based on eqn (1) were 4.6 × 10^3^, 2.3 × 10^4^, 4.8 × 10^4^, and 6.1 × 10^4^, respectively. The intensity of the SERS signal increased as the size of the particles constituting each monolayer increased. On the other hand, the number of hot spots per unit area decreases with an increase in the diameter of the β-CD-capped AuNP monolayer. If the number of hotspots is the main factor in improving the sensitivity of SERS, the smaller β-CD-capped AuNP monolayer was expected to be more favourable for SERS sensing because of the higher hot spot density. However, we observed that the strong electromagnetic fields generated by the large plasmonic NPs are more effective for SERS than the number of hot spots; this trend is similar to previously reported studies using citrate-capped AuNPs.^37,38^ Note that the total surface area of the structure is constant regardless of the particle size, assuming a close-packed structure of true spheres on a two-dimensional plane. Although this assumption is not completely accurate because the particles in the experiment are not true spheres, the contribution of the difference in the total surface area based on the difference in the particle size to the EFs is negligible.^38^ Therefore, it was confirmed that the sensitivity of SERS was significantly improved by using the large-sized β-CD-capped AuNPs synthesized in this experiment.

### Molecular recognition properties of the CD-capped AuNPs

Due to host–guest interactions, β-CDs can recognize and capture trace amounts of hydrophobic molecules dissolved in water. As a result, target molecules can adsorb on the two-dimensional monolayers of β-CD-capped AuNPs when the monolayer-deposited substrates were immersed in the target solution. Drop casting or drying of the target solution was not required to deposit the target molecules on the monolayers. Here, we examined the molecular recognition ability of the α- and β-CD-capped AuNPs. Since the cavity size of β-CDs is larger than that of α-CDs, we expected the molecular recognition abilities of α- and β-CD capped AuNPs to be different.

**Fig. 6:**
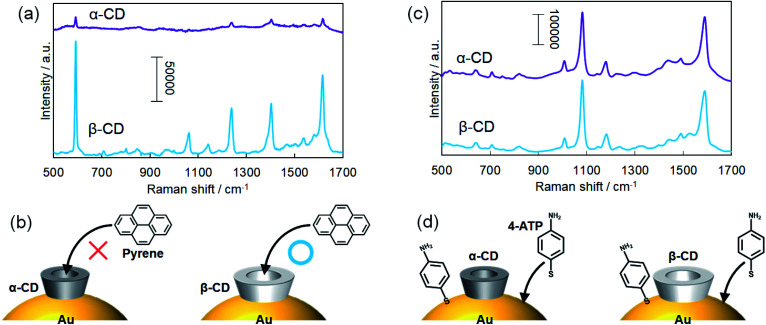
SERS spectra of (a) pyrene and (c) 4-aminothiophenol adsorbed on the monolayers of the α- and β-CD-capped AuNPs. (b) Schematic illustration of α- and β-CD capturing pyrene molecules based on their molecular recognition ability. (d) Schematic of the illustration of 4-ATP adsorption directly on the surfaces of α- and β-CD-capped AuNPs.

A two-dimensional monolayer of α-CD-capped AuNPs was fabricated in the same manner as the two-dimensional monolayers of β-CD-capped AuNPs for SERS sensing. The hydrodynamic diameters of the α- and β-CD-capped AuNPs were measured to be 70 and 74 nm by DLS, respectively. The SEM image of the α-CD-capped AuNP monolayer and its extinction spectrum are shown in Fig. S4 and S5,† respectively. Here, a bulky pyrene molecule was used as a target molecule since the ability of β-CDs to capture pyrene based on host–guest interactions has already been reported.^39^ Pyrene molecules were adsorbed on the α-CD and β-CD-capped AuNP monolayers by immersing the substrates in a pyrene solution. As shown in Fig. 6a, SERS signals were obtained even from the α-CD-capped AuNPs. However, the signal acquired from the β-CD-capped AuNPs was significantly stronger than that obtained from the α-CD-capped AuNPs, indicating that the pyrene recognition ability of the β-CD-capped AuNPs was better than that of the α-CD-capped AuNPs (Fig. 6b). Although the smaller cavity of α-CDs can capture long-chain molecules such as fatty acids,^40^ they do not easily capture sterically bulky molecules. In Fig. 6a, the peak at 593 is assigned to a skeletal stretching mode of pyrene and those at 1239, 1403, and 1616 cm^−1^ are assigned to C–C stretching modes, respectively.^39,41^

Next, 4-ATP was used as a target molecule. As a result, similar SERS signals were obtained from the α- and β-CD-capped AuNPs (Fig. 6c). That result was reasonable because thiol derivatives can directly adsorb on the AuNP surface even when the surface is protected by CD molecules (Fig. 6d). The peaks at 1081 and 1588 cm^−1^ correspond to the vibration modes of aromatic C–S stretching and C–C stretching of 4-ATP, respectively.^42,43^ In this experiment, it was demonstrated that the molecular recognition abilities of α- and β-CD-capped AuNPs are different from each other. Thus, we realized molecular-selective SERS sensing using CD-capped AuNPs.

## Conclusions

In summary, we achieved the size-controlled synthesis of AuNPs modified with CDs, which have a molecular recognition ability. The diameter of the β-CD-capped AuNPs ranged from 24 to 85 nm. As the CD was used as a reducing as well as capping agent in this “green” method, the obtained CD-capped AuNPs were safe and biocompatible. Self-assembled two-dimensional monolayers of closely packed CD-capped AuNPs were also successfully fabricated and applied in SERS sensing. We demonstrated that the larger CD-capped AuNP monolayer (85 nm) exhibited a considerably stronger SERS signal than the smaller CD-capped AuNP monolayer (24 nm) during the detection of R6G. Moreover, it was found that the molecular recognition abilities of the α- and β-CD-capped AuNPs were different from each other. Thus, we realized molecular-selective SERS sensing using the two-dimensional monolayers of CD-capped AuNPs. The results of this study will contribute to the development of not only SERS sensing, but also other applications such as catalysis and self-assembly systems.

## Conflicts of interest

There are no conflicts to declare.

## Supplementary Material

NA-003-D1NA00125F-s001
