# Supplementary material for: Size-controlled synthesis of cyclodextrin-capped gold nanoparticles for molecular recognition using surface-enhanced Raman scattering
Source: Nanoscale Adv. 2021 Apr 7;3(11):3272–8. doi: 10.1039/d1na00125f (PMC9417794; doi:10.1039/d1na00125f)
Supplement: NA-003-D1NA00125F-s001 [file NA-003-D1NA00125F-s001.pdf]

Electronic Supplementary Material (ESI) for Nanoscale Advances.
This journal is © The Royal Society of Chemistry 2021

# Supporting Information

## Size-controlled synthesis of cyclodextrin-capped gold nanoparticles for molecular recognition using surface-enhanced Raman scattering

*Koichiro Saito*, Keegan McGehee, and Yasuo Norikane*

*Email: koichiro.saito@aist.go.jp

*Research Institute for Advanced Electronics and Photonics, National Institute of Advanced Industrial Science and Technology (AIST), Higashi 1-1-1, Tsukuba, Ibaraki 305-8565, Japan.*

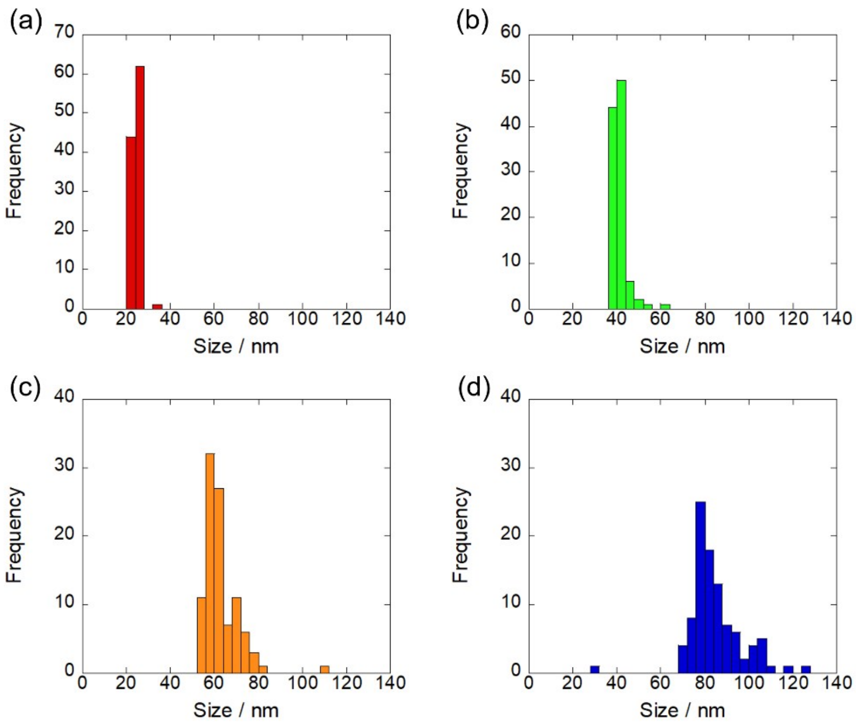


Figure S1. (a, b, c and d) Histograms of size distribution of β-CD-capped AuNPs for each size measured by TEM observation. In order to calculate the average particle size in each of the dispersions, the particle sizes of around 100 particles were measured from TEM images

Table S1. Relation between the particle size, zeta-potential measured by DLS and the amount of seed solution for each AuNPs.

| Volume of added seed solution | Seed | 2 mL | 0.5 mL | 0.2 mL |
|---|---|---|---|---|
| Diameter / Zeta-potential (using α-CD AuNPs) | 35 nm / -44 mV | 45 nm / -43 mV | 70 nm / -44 mV | 84 nm / -44 mV |
| Diameter / Zeta-potential (using β-CD AuNPs) | 28 nm / -44 mV | 37 nm / -45 mV | 55 nm / -46 mV | 74 nm / -48 mV |

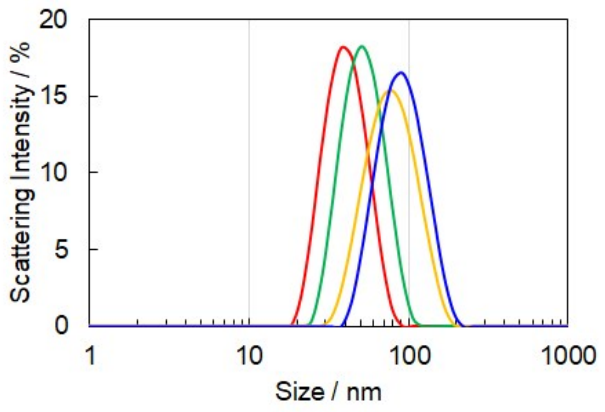


Figure S2. Size distribution of α-CD-capped AuNPs for each size measured by DLS.

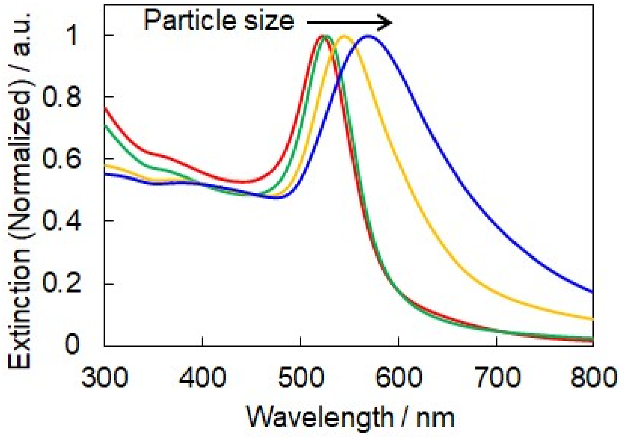


Figure S3. Extinction spectra of the α-CD-capped AuNPs dispersed in an aqueous solution AuNPs.

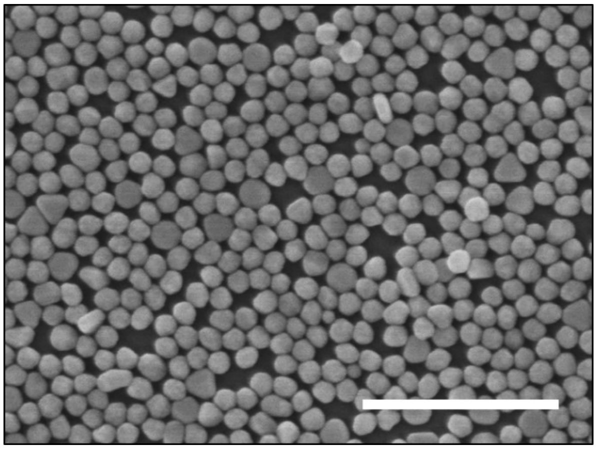

Figure S4. An SEM image of the α-CD-capped AuNP monolayer with diameter of 70 nm measured by DLS. The scale bar is 500 nm.

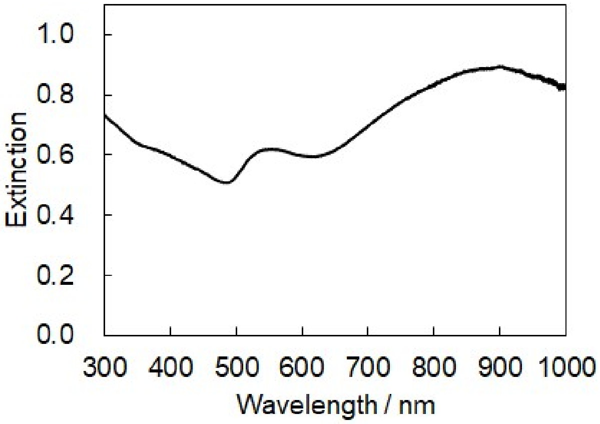


Figure S5. Extinction spectra of the α-CD-capped AuNPs monolayer fabricated on a quartz substrate.
